# Analysis of Mortality and Survival Rate of Liver Cancer in Zhejiang Province in China: A General Population-Based Study

**DOI:** 10.1155/2019/1074286

**Published:** 2019-07-10

**Authors:** Fang-Rong Fei, Ru-Ying Hu, Wei-Wei Gong, Jin Pan, Meng Wang

**Affiliations:** Department of Noncommunicable Control and Prevention, Zhejiang Provincial Center for Disease Control and Prevention, 3399 Binsheng Road, Hangzhou 310051, China

## Abstract

**Background:**

Few accurate up-to-date studies provide liver cancer mortality and survival information in Zhejiang province. This research aimed to depict the mortality and survival of liver cancer in Zhejiang province in China during 2005-2010.

**Methods:**

The data were collected from the Zhejiang Chronic Disease Surveillance Information and Management System, and the mortality rates of liver cancer were calculated by gender, age, and areas. Chinese population census in 2000 and Segi's world population were used for age-standardized mortality rate. The observed and relative survival rates of liver cancer patients were analyzed.

**Results:**

The crude mortality rate of liver cancer was 32.11/10^5^. The age-standardized mortality rate was 17.39/10^5^ and 23.07/10^5^ by Chinese population (ASIRC) and Segi's world population (ASIRW), respectively. The crude liver cancer mortality rate and age-standardized rate in urban areas were lower than those of rural areas. The overall 1-, 3-, and 5-year observed survival (OS) rates of liver cancer patients were 38.61%, 21.65%, and 16.83%, respectively. The 1-, 3-, and 5-year relative survival (RS) rates of liver cancer patients were 39.49%, 23.27%, and 19.09%, respectively. Survival rate decreased obviously within 1 to 5 years and then leveled off. It was shown that the male overall survival rate was higher than the female one and the difference was statistically significant (P<0.05).

**Conclusions:**

Both lower mortality and better survival rates were observed in urban areas, compared to rural areas. Relevant parties including government, public resource, and propaganda department should devote enough attention to rural areas.

## 1. Introduction

In the past few decades, China has experienced a remarkable economic growth accompanied by an epidemiological and demographic transition [1, 2]. Cancer has become the leading cause of death in Zhejiang province in China [3]. Liver cancer is the second most common cause of cancer deaths of the residents in Zhejiang province. As a result of severe environmental pollution and shifting lifestyle, liver cancer mortality increases recently. According to the GLBOCAN 2012 report [4], an estimated 745,500 deaths occurred worldwide during 2012, with China alone accounting for about 50% of total number of cases and deaths. Majority of studies investigating liver cancer in China are clinical-related, such as diagnosis, molecular pathogenesis, and treatments [5–9]. Only few epidemiological studies show liver cancer survivorship in China, and large population-based studies were seldom conducted. Zhejiang province, one of the most developed and wealthiest provinces, lies in the southern China with a population of 45 million. This study is to explore the descriptive analysis and survivorship of liver cancer in Zhejiang, Chana, from 2005 to 2010.

## 2. Methods

### 2.1. Data Source

The population-based cancer surveillance in Zhejiang was established in January 2001 to obtain reliable incidence and mortality data on cancer among the Zhejiang residents. The data was collected from the Zhejiang Chronic Disease Surveillance Information and Management System during 2005-2010, which was published by Zhejiang Provincial Center for Disease Control and Prevention (CDC). Among 90 districts in Zhejiang province, 30 districts, including 12 urban areas and 18 rural areas, were selected as the surveillance regions to represent the entire province, accounting for variations of geography and socioeconomic status. A prospective population-based death cause registry system, maintained by Zhejiang Provincial CDC, was available in these selected districts, covering 36.9% of population (16.6 million) in Zhejiang province. Data on the population in each district was provided by the Bureau of Statistics. Liver cancer was defined by C22 coding by the International Classification of Diseases, the 10th edition. The percentage of hepatocellular carcinoma within all liver tumors was 92.73%.

### 2.2. Quality Control

The cancer data were collected according to the requirements of the “Chinese Guideline for Cancer Registration” [10] and “Cancer Incidence in Five Continents Volume IX” [11] by the International Agency for Research on Cancer/International Association of Cancer Registration (IARC/IACR). These criteria to assure the quality of data included only areas with the proportion of morphological verification (MV%) higher than 66%, percentage of cancer cases identified with death certification only (DCO%) less than 15%, and mortality to incidence ration (M/I), which proved to be an effective indicator of disparities in cancer screening, treatment, and survival, between 0.6 and 0.8.

### 2.3. Data Analysis

Registry data of cancer death and follow-up from Zhejiang province were used to calculate the crude mortality rate, age-specific mortality rate (grouped by 0, 1-4, 5-9,…, 80-84, 85 years old and above), and liver cancer mortality rate. All rates were presented as per 100,000 person-years in this study [12]. The Chinese population census in 2000 and the population of Segi were used to obtain age-standardized rates (ASR) of mortality, truncated mortality rate, and observed and relate survival rate of liver cancer. Z-test and *χ*^2^-test were used to compare survival rate. Analysis was conducted using SAS 9.3, Excel 2010, and SRUV3.01 software.

## 3. Results

### 3.1. Overall Mortality Rate

A total of 24,341 cases of liver cancer were registered in the 6-year period between 1st January 2005 and 31st December 2010. 18,207(74.80%) cases were males and 6,134(25.20%) were females. 7,824 (32.14%) liver cancer patients lived in urban areas and 16,517 (67.86%) lived in rural areas. The crude mortality rate of liver cancer was 32.11/10^5^ (47.39/10^5^ in males and 16.40/10^5^ in females). The age-standardized mortality rate was 17.39/10^5^ and 23.07/10^5^ by Chinese population (ASIRC) and Segi's world population (ASIRW), respectively. Among patients aged 0-74 years, the cumulative mortality rate was 2.62%, whereas truncated rate was 42.62/10^5^ in patients aged 35-64 years. Both crude liver cancer mortality rate and age-standardized rate in urban areas were lower than those of rural areas. The overall mortality rates are shown in Table 1.

### 3.2. Age-Specific Mortality Rates

**Figure 1:**
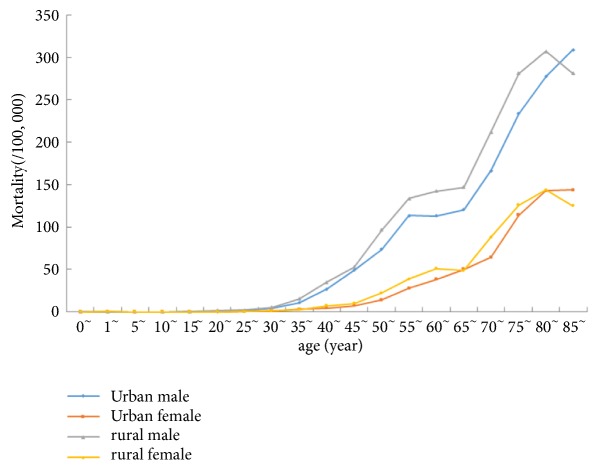
Age-specific incidence rate of liver in Zhejiang urban and rural areas from 2005 to 2010.

Figure 1 shows the age-specific mortality rate curves stratified by sex and area types (urban/rural). Liver cancer mortality was relatively low before 40 years old, increased dramatically after 40 years old, peaked after 80 years old, and then slightly decreased after 80 years old. The mortality rate curves were similar between urban and rural areas except that urban male mortality rate curve continued to increase after 80 years old. Comparing the age-specific mortality rate between urban and rural areas, we found that the mortality rate in males was lower in urban areas than in rural areas in most age groups except for age groups 1, 5, 10, and over 80 years. Similarly, the mortality rate in females was higher in rural areas than in urban areas for the majority age groups, except for the age groups before 15 years old.

### 3.3. Observed and Relative Survival Rate of Liver Cancer

The overall 1-, 3-, and 5-year observed survival (OS) rates of liver cancer patients were 38.61%, 21.65%, and 16.83%, respectively. The 1-, 3-, and 5-year relative survival (RS) rates of liver cancer patients were 39.49%, 23.27%, and 19.09%, respectively. The 5-year OS rates were 17.21% in males and 15.66% in females, and the 5-year RS rates were 19.45% in males and 17.97% in females (Table 2). The OS and RS rates decreased with the increase of survival time in male or female liver cancer patients. Survival rate decreased obviously within 1 to 5 years and leveled off afterwards. It was shown that the male overall survival rate was higher than that of females and the difference was statistically significant (*χ*2=6.31, P<0.05).

### 3.4. Survival Rate of Regional Distribution

The 1-, 3-, and 5-year RS rates were 46.34%, 31.09%, and 26.91% (46.69%, 31.59%, and 27.16% in males, respectively; 45.26%, 29.56%, and 26.14% in females, respectively) in urban areas. The 1-, 3-, and 5-year RS rates were 36.47%, 19.84%, and 15.72% (36.70%, 20.27%, and 16.14% in males, respectively; 35.74%, 18.49%, and 14.39% in females, respectively) in rural areas. The RS rate decreased with the increase of survival time in urban and rural (Table 3). The RS rate of urban was higher than the rural one and the difference was statistically significant (*χ*2=288.06, P<0.05).

### 3.5. The 5-Year Survival Rate by Age Group

The 5-year OS and RS rates decreased as age increased. The 5-year OS and RS rates were the highest at 15-44 age group compared to other age groups in males and females, 21.76%, 21.94% and 23.29%, 23.38%, respectively. The 5-year OS and RS rates were the lowest at 75 age group in males and females, 14.22%, 22.83% and 11.03%, 16.45%, respectively (Table 4).

## 4. Discussion

This population-based cancer surveillance system was designed not only for control planning, implementation, and evaluation on cancer prevention and control, but also for scientific research [13]. Data from cancer patients has been collected according to the data collection criteria to assure its quality since 2001 in these Zhejiang surveillance areas. Therefore, this cancer data is complete, valid, and reliable.

The Zhejiang overall crude mortality of liver cancer (32.11/10^5^) was higher than the national crude mortality of liver cancer in China (24.70/10^5^), assessed by the National Cancer Center [14]. The ASMRW mortality rate (23.07/10^5^) of Zhejiang was higher than the world average (9.50/10^5^). Zhejiang province had a similar trend of age-specific mortality rates to the one in China [13]. Some research had shown that people infected with HBV over six months can develop chronic infection, with some developing liver cancer [14]. In adults with chronic HBV infection, the proportion of HCC development is about 5% every 10 years, which is from 100 to 300 times the probability of HCC development in those who are not infected with HBV [14]. About 300,000 people die, who contributed to HBV infection each year, and 50% of whom die from HCC, most of which are related to HBV infection [15].

Both crude liver cancer mortality rate and age-standardized rate in urban areas were lower than those of rural areas. This discrepancy in liver cancer mortality in Zhejiang was consistent with the findings of the National Cancer Center [16]. The difference in survival might be due to the disparity in health care accessibility, including treatment availability, between urban and rural areas [17], which could be caused by multiple reasons. First, urban residents might pay more attention to their health than those residing in rural areas. Moreover, public health resources are more available in urban areas than in rural areas. In Zhejiang province, urban residents would have a higher socioeconomic status than rural residents. Additionally, according to the current knowledge of liver cancer etiology, the main risk factors, such as HBV infection, aflatoxin contamination, and HCV infection, are more prevalent in rural areas [18].

The present survival rate is calculated mainly based on the clinical data, which could be confounded by many factors, such as medical level, treatment method, and the source of patients. Relative survival rate was estimated by taking account of the characteristics of the general population (the same period, the same sex, and the same age group). As a result, the impact of these factors (age, gender, diagnosis year, and other factors) was eliminated, and the RS rate was commonly used in survival analysis of different regions. Our study showed that the 5-year RS rate of liver cancer in Zhejiang province was 19.09%, which was higher than the RS rate reported in Qidong, Jiangsu province, during 2001-2007 [19]. This may be related to the period of analysis, which was different. Furthermore, the level of economic development between two study regions was different. As for the developed countries, the 5-year RS rate in Japan had exceeded the level of our liver cancer survival rate in the 1997-1999 years [20]. However, the 5-year RS rate in the Republic of Korea was 25.1% in 2005-2009, which was significantly higher than that in Zhejiang province [21]. In the United States, the 5-year RS rate of liver cancer was 10.0% between 1973 and 2003 [22]. The better survival in these developed countries might be due to different criteria for malignant tumor diagnosis, indicating that the early detection and the better treatment of liver cancer are needed in China. Age might be another factor contributing to the differences in the survival rate of liver cancer. In our study, 5-year OS rate of young (15-34 years old) cases was 22.01%, which was better than that in elderly patients (>75 years old) which was 13.08%. However, the lowering OS rate among older patients might be misleading, because of other competing risks of mortality after liver cancer diagnosis. When considering the probability of survival of the population, the RS rate of the elderly was not lower than that of the young.

The results of this study showed that rural residents had both a higher mortality rate and a worse survival rate of liver cancer, compared to those living in the urban areas. This could result from the low socioeconomic status (SES), the delayed treatment, medical insurance system, health education, and other factors. Several different researches showed that SES, medical resource allocation, the treatment of timeliness, medical insurance system, and education affected the survival rate of liver cancer patients [23–26]. Therefore, more attention should be drawn to rural areas for liver cancer prevention. In particular, early stage of liver cancer screening must be developed and implemented in rural areas. Appropriate allocation of resources for cancer prevention, early diagnosis, and curative and palliative care require detailed knowledge of the local burden of liver cancer [27, 28].

## 5. Conclusions

Population-based cancer surveillance data is a valuable resource for liver cancer research. We have conducted a mortality and survival study by gender, age at diagnosis, and residential areas (urban/rural), which has not been done in Zhejiang province in China. This study suggested that both a lower mortality rate and a better survival rate were observed among urban residents, compared to rural residents.

## Figures and Tables

**Table 1:** The incidence and mortality rates of live cancer in Zhejiang province and their ranking among all cancers from 2005 to 2010.

	Crude rate (1/10^5^)	ASR (China)	ASR (Word)	Cumulated rate (0-74 years)/%	Truncated rate (35-64 years) (1/10^5^)	Ranking
Incidence						
Total						
Both Sexes	31.40	16.64	21.97	2.51	40.99	3
Male	46.56	25.23	33.24	3.76	63.70	3
Female	15.91	7.87	10.60	1.21	16.91	5
Urban						
*Both Sexes*	27.64	14.14	18.88	2.14	33.23	4
Male	40.56	21.19	28.32	3.16	51.44	3
Female	14.62	7.00	9.51	1.08	14.15	6
Rural						
*Both Sexes*	33.18	17.86	23.47	2.69	44.74	3
Male	49.38	27.18	35.60	4.05	69.58	3
Female	16.53	8.29	11.12	1.27	18.25	5
Mortality						
Urban and rural						
*Both Sexes*	32.11	17.39	23.07	2.62	42.62	2
Male	47.39	26.39	34.90	3.94	66.61	2
Female	16.40	8.18	11.10	1.25	17.09	3
Urban						
*Both Sexes*	29.93	15.14	20.22	2.25	36.39	2
Male	44.13	23.05	30.68	3.41	58.07	2
Female	15.48	7.11	9.74	1.06	13.68	3
Rural						
*Both Sexes*	33.25	18.67	24.67	2.83	46.14	2
Male	49.09	28.25	37.24	4.23	71.36	2
Female	16.89	8.81	11.89	1.36	19.07	3

**Table 2:** Observed and relative survival rate of liver cancer in Zhejiang province from 2005 to 2010 (%).

Survival time (year)	Male		Female		Total	
	OS	RS	OS	RS	OS	RS
1	38.89	39.75	37.76	38.70	38.61	39.49
2	27.54	28.82	25.42	26.74	27.03	28.31
3	22.11	23.70	20.26	21.90	21.65	23.27
4	19.03	20.93	16.87	18.78	18.50	20.41
5	17.21	19.45	15.66	17.97	16.83	19.09
6	15.64	18.18	14.35	17.00	15.33	17.89
7	14.63	17.50	13.95	17.06	14.46	17.40

**Table 3:** Relative survival rate of liver cancer in Zhejiang province from 2005 to 2010 (%).

Survival time (year)	Urban			Rural		
	Male	Female	Total	Male	Female	Total
1	46.69	45.26	46.34	36.70	35.74	36.47
2	36.27	34.31	35.78	25.56	23.34	25.02
3	31.59	29.56	31.09	20.27	18.49	19.84
4	28.53	27.36	28.24	17.65	15.03	17.00
5	27.16	26.14	26.91	16.14	14.39	15.72
6	25.14	24.99	25.11	15.19	13.51	14.79
7	24.33	25.37	24.61	14.58	13.43	14.31

**Table 4:** The 5-year relative survival at age group in Zhejiang province from 2005 to 2010(%).

Age (year)	Male		Female		Total	
	OS	RS	OS	RS	OS	RS
15-44	21.76	21.94	23.29	23.38	22.01	22.18
45-54	17.88	18.31	21.87	22.11	18.55	18.96
55-64	17.69	18.62	18.03	18.53	17.78	18.61
65-74	15.97	18.53	13.23	14.56	15.22	17.41
≥75	14.22	22.83	11.03	16.45	13.08	20.44

## Data Availability

The data are available from the corresponding author upon request.
